# Successful Use of Recombinant Activated Factor VII to Reverse Ticagrelor-Induced Bleeding Risk: A Case Report

**DOI:** 10.1055/s-0038-1672211

**Published:** 2018-09-27

**Authors:** Anne Godier, Mélanie Dupont, Jean-Philippe Desilles, Caroline Le Guerinel, Guillaume Taylor, Mathilde Perrin, Anne-Céline Martin, Pascale Gaussem

**Affiliations:** 1 Service d'Anesthésie-Réanimation, Fondation Adolphe de Rothschild, Paris, France; 2 Sorbonne Paris Cité, Faculté de Pharmacie, Université Paris Descartes, Paris, France; 3 INSERM UMR-S1140, Paris, France; 4 Département de Neuroradiologie Interventionnelle, Fondation Adolphe de Rothschild, Paris, France; 5 Sorbonne Paris Cité, Laboratory of Vascular Translational Science, Université Paris Diderot, Paris, France; 6 INSERM UMR 1148, Paris, France; 7 Département de Neurochirurgie, Fondation Adolphe de Rothschild, Paris, France; 8 Service de Cardiologie, Service de Santé des Armées, Hôpital d'Instruction des Armées Percy, Clamart, France; 9 Service d'hématologie Biologique, AP-HP, Hôpital Européen Georges Pompidou, Paris, France

**Keywords:** antithrombotic, platelet transfusion, recombinant activated factor VII, reversal, ticagrelor

## Abstract

Management of ticagrelor-associated bleeding is challenging, especially in neurosurgery. Platelet transfusion is inefficient and no antidote is currently available. We report here the first case of recombinant activated factor VII (rFVIIa) use to bypass ticagrelor-induced platelet inhibition. A woman treated with ticagrelor and requiring emergent neurosurgery for an intracranial hematoma received preoperative high-dose platelet transfusion and 60 μg/kg rFVIIa. Laboratory monitoring demonstrated that platelet transfusion failed to reverse ticagrelor-induced platelet inhibition while rFVIIa improved hemostasis by shortening the thromboelastometric clotting time. Neurosurgery occurred without any bleeding event but the patient presented with a postoperative pulmonary embolism. In conclusion, rFVIIa may decrease ticagrelor-induced bleeding risk but careful assessment of the benefit-risk balance is warranted before using rFVIIa to reverse ticagrelor effects.

The major safety issue with antiplatelet agents is bleeding. Ticagrelor is an orally available, direct-acting, selective, and reversibly binding P2Y _12_ receptor antagonist approved as first-line therapy for the treatment of acute coronary syndrome. 1 Management of ticagrelor-associated bleeding is challenging, especially in neurosurgery: platelet transfusion, usually recommended to reverse antiplatelet agents, is inefficient; desmopressin is unlikely to be an effective therapeutic agent for control of ticagrelor-associated bleeding risk; and no specific antidote is currently available. 2 3 The summary of product characteristics for ticagrelor suggests that recombinant activated factor VII (rFVIIa) “may increase hemostasis” (although unlabeled use). 1 Indeed, rFVIIa is a potent hemostatic bypassing agent that boosts thrombin generation by massive activation of the extrinsic pathway of the coagulation cascade. 4 It is approved for the treatment of bleeding disorders including hemophilia with inhibitors but also Glanzmann's thrombasthenia with antibodies to glycoprotein IIb–IIIa or human leukocyte antigen. It has previously been shown to reduce clopidogrel-enhanced blood loss after punch biopsy in healthy subjects. 5 An in vitro study using blood samples spiked with ticagrelor reported that rFVIIa accelerates thrombin generation, and thus clot formation, and therefore may decrease ticagrelor-induced bleeding as observed in a mouse model. 6 7 However, no clinical data support rFVIIa efficacy to reverse ticagrelor. We report here the first case of rFVIIa used to bypass ticagrelor-induced platelet inhibition with a good clinical outcome. The patient gave informed consent for the publication of this case report.

A 58-year-old woman underwent an elective endovascular stent-assisted coiling of a right unruptured middle cerebral artery aneurysm after a 24-hour premedication with dual antiplatelet therapy combining ticagrelor 90 mg twice daily and aspirin 250 mg per day. Aneurysm perforation occurred during coil deployment, inducing intracranial bleeding with intracerebral hematoma, intracranial hypertension, and mass effect, eventually followed with stent thrombosis. Emergent decompressive craniectomy and hematoma removal were needed. Classified as high bleeding risk surgery as all neurosurgical procedures, this procedure required careful preoperative correction of hemostasis, and international guidelines propose to correct platelet function in patients with aspirin- or P2Y _12_ receptor antagonists–associated intracranial hemorrhage before neurosurgical procedures. 8

To reach this aim, platelet transfusion and rFVIIa administration were proposed. High-dose platelet transfusion was performed using two ABO-compatible, irradiated, pooled, random-donor platelet concentrates, stored for 2 days, resulting in 9.9 × 10 ^11^ transfused platelets, equivalent to 20 platelet units (PU) (irradiated platelets were oddly provided as such by the blood bank but unrelated to the patient status). The total amount of transfused platelets represents 2- to 2.5-fold the French recommended dose for a 80-kg patient (recommended dose of 0.5 to 0.7 × 10 ^11^ platelets/10 kg). 9 Platelet transfusion was immediately followed by the administration of 60 μg/kg rFVIIa; then, the patient was transferred straightaway to the operating room. 10 Craniectomy and hematoma removal were performed, the surgery lasted 1.5 hours, no bleeding complication occurred, and hemostasis was reported as normal by the neurosurgeon. The postprocedural cerebral computed tomography showed no further hemorrhage but a thoracic computed tomography performed to detect potential rFVIIa-associated thrombotic events revealed a segmental pulmonary embolism, requiring progressive therapeutic anticoagulation with heparin and then with vitamin K antagonists. Three months later, the patient was discharged, conscious but with residual left hemiplegia related to the ischemic stroke downstream stent thrombosis.

Fig. 1 summarizes platelet and coagulation monitoring during hemostatic treatment. Monitoring included (1) platelet count, (2) VerifyNow (Accumetrics, San Diego, California, United States), a standardized point-of-care device that assesses platelet reactivity to antiplatelet agents on whole blood with two different cartridges, one for aspirin and one for P2Y _12_ receptor antagonists, 11 (3) flow cytometric assay for platelet vasodilator-associated stimulated phosphoprotein (VASP) phosphorylation to specifically monitor P2Y _12_ receptor inhibition (Becton Dickinson, Le Pont-de-Claix, France), 11 and (4) rotational thromboelastometry (ROTEM, Werfen, Barcelona, Spain) to assess the extrinsic pathway of coagulation (EXTEM test), especially the clotting time, defined as the time to clot initiation and the clot firmness. 12 Before platelet transfusion, i.e., 14 hours after last antiplatelet therapy intake, platelet inhibition in response to aspirin (VerifyNow-Aspirin) and ticagrelor (VerifyNow-P2Y _12_ and VASP) was present with respective values below the admitted platelet reactivity thresholds for antithrombotic efficacy. 11 13 Since the patient received only two doses of ticagrelor before the procedure, ticagrelor-induced platelet inhibition was lower than usually described in case of long-term therapy. 14 The transfusion of 10 PU reversed the effect of aspirin as shown by the leap of VerifyNow-Aspirin result above the threshold value. The total 20 PU (9.9 × 10 ^11^ transfused platelets) increased platelet count with a percentage of platelet recovery approaching 100% but failed to reverse ticagrelor-induced platelet inhibition, as both VASP and VerifyNow-P2Y _12_ values remained below thresholds. rFVIIa had no effect on platelet function but improved hemostasis by shortening the EXTEM clotting time, whereas maximum clot firmness remained unchanged.

**Fig. 1:**
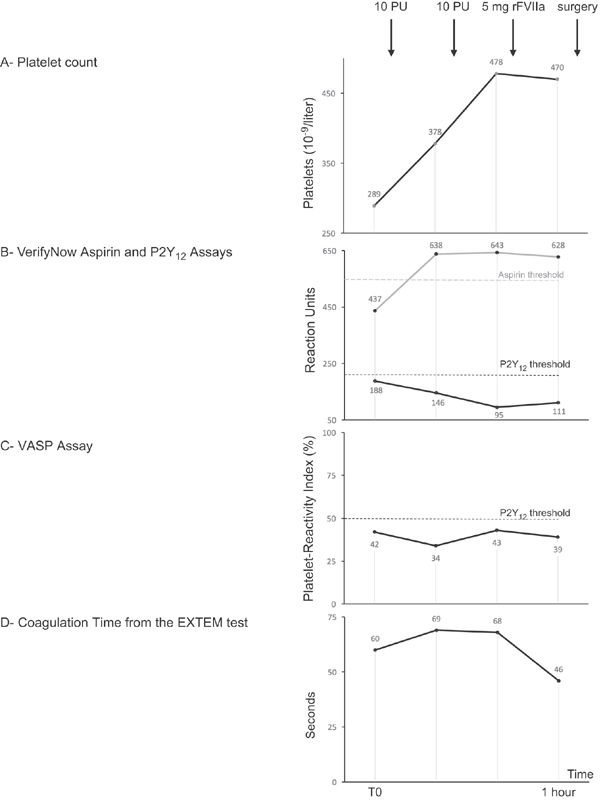
Platelet and coagulation monitoring during hemostatic treatment. Monitoring included platelet count (10 ^9^ /L), VerifyNow-Aspirin (aspirin reaction units), VerifyNow-P2Y _12_ (P2Y _12_ reaction units), VASP assay (platelet reactivity index, %), and EXTEM test from the ROTEM device (clotting time, seconds). Dashed lines show platelet reactivity thresholds for antithrombotic efficacy for each test. 11 13 PU: platelet unit (1 PU is equivalent to 0.5 × 10 ^11^ transfused platelets; thus, the total dose of transfused platelets was 9.9 × 10 ^11^ platelets); rFVIIa: recombinant activated factor VII; T0: time immediately before platelet transfusion.

This observation confirms that platelet transfusion, as already well documented, readily and promptly corrects aspirin effects. On the contrary, high-dose platelet transfusion is inefficient to reverse ticagrelor, as previously reported in a few case reports 15 16 17 18 19 and a small case series. 3 Indeed, circulating ticagrelor and its active metabolite, present at high plasma concentrations, immediately inhibit the transfused platelets. Moreover, this case suggests that rFVIIa may bypass ticagrelor effects by boosting coagulation: rFVIIa binds to tissue factor, increases thrombin generation, and improves hemostasis, and thus it may overcome platelet inhibition. 20 Although this report only suggests a relationship between rFVIIa and bleeding control, rFVIIa currently appears as the only potential option to improve hemostasis in ticagrelor-treated patients facing severe bleeding, especially when emergent neurosurgery is needed. We hypothesized that rFVIIa alone would result in the same hemostasis improvement; therefore, platelet transfusion, although performed here to comply with previous guidelines, may not be necessary. Specific tests performed here to assess respective effects of both platelet transfusion and rFVIIa on hemostasis did not impact therapeutic strategy. Thus, they do not seem to be required before rFVIIa administration in such a context. Last, this observation also underlines that thromboembolism is a common and potentially serious side effect of rFVIIa, which should be closely monitored. 21

In conclusion, rFVIIa may decrease ticagrelor-induced bleeding risk but careful assessment of the benefit–risk balance is warranted before using rFVIIa to reverse ticagrelor effects.
